# Chitosan Nanoparticles as a Biostimulant During In Vitro Multiplication of Vanilla Using Temporary Immersion Bioreactors

**DOI:** 10.3390/molecules31020328

**Published:** 2026-01-18

**Authors:** Víctor Adrián Delgado-Rivera, María Karen Serrano-Fuentes, José María Rivera-Villanueva, Juan Antonio Pérez-Sato, Jericó Jabín Bello-Bello

**Affiliations:** 1 Colegio de Postgraduados-Campus Córdoba, Carretera Federal Córdoba-Veracruz km. 348, Amatlán de los Reyes 94953, Veracruz, Mexico; victor_adr1999@hotmail.com (V.A.D.-R.); ame_karen15@hotmail.com (M.K.S.-F.); pantonio@colpos.mx (J.A.P.-S.); 2 Facultad de Ciencias Químicas, Universidad Veracruzana, Prolongación de Oriente 6, No. 1009, Col. Rafael Alvarado, Orizaba 94340, Veracruz, Mexico; chemax7@yahoo.com.mx; 3 SECIHTI-Colegio de Postgraduados-Campus Córdoba, Carretera Federal Córdoba-Veracruz km. 348, Amatlán de los Reyes 94953, Veracruz, Mexico

**Keywords:** hormesis, chlorophyll, phenolic content, lipid peroxidation, antioxidant

## Abstract

This research aimed to assess the effect of chitosan nanoparticles (ChNPs) during in vitro shoot proliferation of vanilla using temporary immersion bioreactors (TIB). TIB culture is a biotechnological process that uses semiautomated containers for the production of explants exposed in liquid culture medium. Concentrations of control, 25, 50, 100, 200, and 400 mg/L ChNPs were evaluated in Murashige and Skoog culture medium. Morphological characterization of ChNPs was performed using scanning electron microscopy. At 60 days of culture, survival (%), development variables, photosynthetic pigment content, lipid peroxidation expressed in malondialdehyde, total phenolic content (TPC), hydrogen peroxide (H_2_O_2_) content, and total antioxidant capacity (TAC) expressed in trolox equivalents were evaluated. The data were analyzed with analysis of variance, with a Tukey test (*p* ≤ 0.05) using SPSS statistics software, version 29. The results revealed that the greatest survival (%) was obtained at concentrations of control, 25, and 50 mg/L ChNPs, while the lowest survival (%) was observed at concentrations of 400 mg/L ChNPs. Growth stimulation was found, as well as an increase in chlorophyll and *β*-carotene at concentrations of 25 and 50 mg/L ChNPs. The level of H_2_O_2_ increased at 25 and 50 mg/L ChNPs. Lipid peroxidation showed no differences among treatments. TPC increased at 100 and 200 mg/L ChNPs, while TAC increased at 200 and 400 mg/L ChNPs. In conclusion, the administration of ChNPs at low concentrations can stimulate growth, while at high concentrations they can inhibit it, a response known as hormesis or hormetic effect.

## 1. Introduction

Nanoscale materials are defined as materials having at least one of their dimensions on a nanometric scale (1–100 nm). This scale can be presented in their length, height, or depth and depending on the number of dimensions they are classified as zero-dimensional nanomaterials (quantum dots, hollow spheres, fullerenes, and nanoparticles), one-dimensional nanomaterials (nanofibers, nanotubes, nanowires, and nanorods), two-dimensional nanomaterials (nanolayers, nanofilms, and nanoplates), and three-dimensional nanomaterials (bulk powders, nanoparticle dispersions, and polycrystals) [1,2]. Nanoparticles (NPs) have applications in the energy industry, medicine, cosmetology, and agriculture [3]. In agriculture, nanomaterials such as silver nanoparticles, zinc oxide nanoparticles, gold nanoparticles, copper nanoparticles, cobalt nanoparticles, and chitosan nanoparticles (ChNPs) have been used [4]. Chitosan is a biodegradable biopolymer obtained from the deacetylation of chitin; it is non-toxic at low concentrations and stimulates plant defense mechanisms [5]. It has microbicidal activity [6] and has been reported to have a biostimulant effect on in vitro shoot proliferation during micropropagation [3]. The main advantages of micropropagation are the homogeneous and continuous production of plantlets in a laboratory, requiring only a short time, that is not affected by regional or seasonal [7]. However, traditional micropropagation in semisolid medium is slow, has an insufficient proliferation rate, and the gelling agents significantly increase the cost and limit the ability to automate mass micropropagation; on the other hand, in vitro propagation using TISs in liquid medium provides a larger multiplication rate in less time, combines aeration and explant immersion for the semiautomation of the in vitro culture process, and reduces the cost of plantlet production compared to the conventional method in semisolid culture medium [8]. To date, there is no evidence of the application of chitosan in vanilla during ex vitro or in vitro conditions. Different studies have demonstrated the efficiency of chitosan administration during in vitro multiplication of indian borage (*Coleus aromaticus*), morning glory (*Ipomea purpurea*), and flossflower (*Ageratum houstonianum*) [9,10,11]. However, the use of chitosan at the nanometric scale can increase its biostimulant effects, also known as the hormetic effect or hormesis, which consists of a biphasic response caused by a stressor, which at a low concentration stimulates development, while at a high concentration it can inhibit development or cause the death of the explants [12].

Vanilla (*Vanilla planifolia* Jacks. ex Andrews) is an orchid of economic, cultural, and ecological importance thanks to vanillin, a natural compound present in its fruits and of great interest to the food, cosmetics, and pharmaceutical industries [13]. The annual production of natural vanillin is approximately 45–50 metric tons per annum [14]. Mexico contributes less than 1% of global vanilla production. In recent years, national production has reached 589 tons [15,16]. According to Food and Drug Administration (FDA) regulation, the vanillin can be labeled as “natural vanillin” only if the vanillin comes from vanilla beans. Therefore, any vanillin that does not come from vanilla beans cannot be called “natural” [17]. Although Mexico is the center of origin of vanilla, the crop presents problems due to the low germination rate of its seeds and the fact conventional propagation by cuttings is slow, there is no rejuvenation of plantations, it can transmit pests and diseases, and there is limited availability of plant material. In addition, vanilla is in the category subject to special protection by NOM-059 [18] and internationally it is currently classified in category B2ab (iii, v) “Endangered” in the Red List of Threatened Species [19]. Due to the importance of this species, micropropagation is a biotechnological alternative that allows the in vitro regeneration of elite plants free of pests and diseases [20]. The use of temporary immersion bioreactors (TIBs) has managed to reduce the costs and time of plant regeneration by increasing the multiplication rate [7]. The use of ChNPs using TIBs in vanilla could increase the in vitro multiplication rate. Thus, we hypothesize that the combination of ChNPs and TISs could have a synergistic effect to improve physiological development and can be applied to micropropagation systems. This work aimed to evaluate the biostimulant effect of ChNPs during in vitro shoot proliferation of *Vanilla planifolia* through temporary immersion bioreactors.

## 2. Results

### 2.1. SEM Analysis of ChNPs

**Figure 1:**
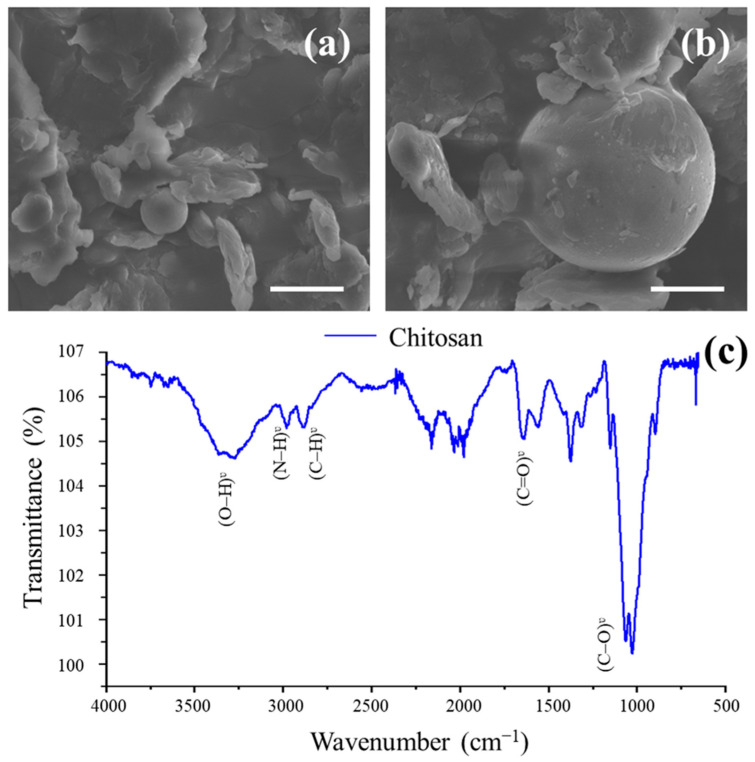
Scanning electron microscopy analysis of ChNPs. (**a**) White bar = 150 nm, (**b**) white bar = 50 nm, (**c**) Fourier Transform Infrared Spectroscopy spectrum of the chitosan molecule highlighting the characteristic absorption bands of its principal functional groups. ChNPs: chitosan nanoparticles.

Physical characterization by SEM corresponds to the mark, with a white appearance and diameter of 80–100 nm (Figure 1a,b).

### 2.2. Fourier Transform Infrared Spectroscopy

FT⎼IR spectroscopy shows a strong band in the 3370–652 cm^−1^ region corresponding to hydroxyl (O⎼H) and amine group (N⎼H) stretching, as well as the intramolecular hydrogen bonds. The absorption peak at around 2886 cm^−1^ can be related to alkyl group (C⎼H). N⎼acetyl groups (CH_3_CO) were confirmed by the band at around 1654 cm^−1^ from amide (C=O) and 1316 cm^−1^ from amide (C⎼N), respectively. A peak at 1563 cm^−1^ corresponds to the amine group (N⎼H) bending of the primary amine. The methylene group (CH_2_) bending and methyl group (CH_3_) symmetrical deformations were verified by the presence of bands at around 1428 and 1375 cm^−1^, respectively. The absorption peak at 1152 cm^−1^ can be related to asymmetric stretching of the ether group (C⎼O⎼C) bridge. The peaks at 1067 and 1029 cm^−1^ correspond to hydroxyl group (C⎼O) stretching (Figure 1c).

### 2.3. Effect of ChNPs on Shoot Proliferation

**Figure 2:**
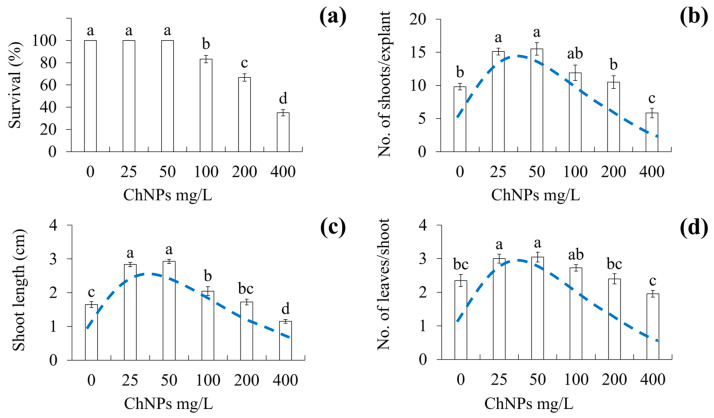
Effect of ChNPs on the in vitro shoot proliferation of vanilla (*Vanilla planifolia* Jacks. ex Andrews). (**a**) Survival, (**b**) shoots per explant, (**c**) shoot length, (**d**) leaves per shoot after 60 days of culture in vitro in a temporary immersion bioreactor. Data are expressed as mean values ± standard error (SE) to represent central tendency and variability. Means with a different letter indicate statistical significance (Tukey, *p* ≤ 0.05). ChNPs: chitosan nanoparticles; blue dashed line: hormetic dose–response curve depicting low-dose stimulatory (peak) and high-dose inhibitory responses (decline).

**Figure 3:**
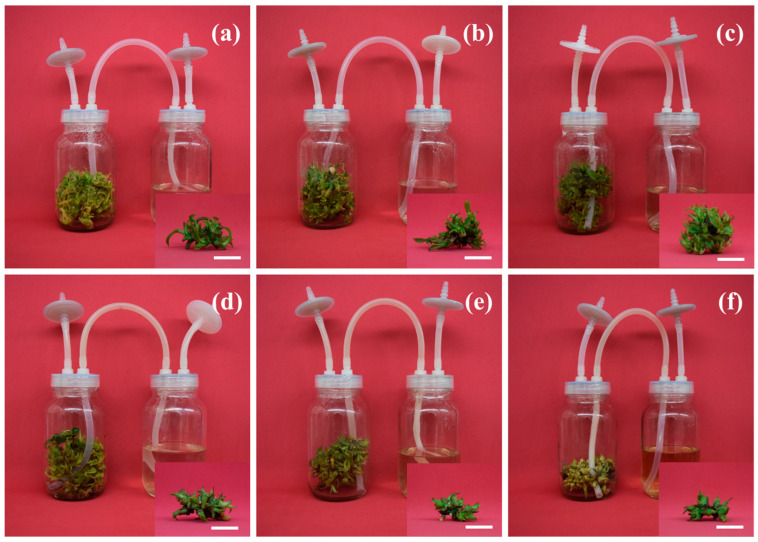
Effect of ChNPs on in vitro shoot proliferation of vanilla (*Vanilla planifolia* Jacks. ex Andrews) after 60 days of culture in temporary immersion systems (TIB). (**a**–**f**) Control, 25, 50, 100, 200, and 400 mg/L ChNPs, respectively. ChNPs: chitosan nanoparticles. White bar = 5 cm.

The application of different ChNP concentrations showed an effect on the survival (%), and development variables in vanilla shoot culture in a temporary immersion bioreactor (Figure 2). The highest survival (%) was obtained at concentrations of control, 25, and 50 mg/L ChNPs, with 100% survival, while the lowest survival (%) was obtained at concentrations with 400 mg/L ChNPs, with 35% survival. For the number of shoots per explant, the greatest number was found at concentrations with 25 and 50 mg/L ChNPs, with 15.10 and 15.50 shoots per explant, respectively, while the lowest number of shoots was found at concentrations of 400 mg/L ChNPs, with 5.83 shoots per explant. The greatest shoot height was found at concentrations of 25 and 50 mg/L ChNPs, of 2.83 and 2.92 cm length, respectively, while the shortest shoot length was obtained at concentrations of 400 mg/L ChNPs, of 1.15 cm length. The highest number of leaves per shoot was observed at concentrations of 25 and 50 mg/L ChNPs, with 3.00 and 3.04 leaves per shoot, respectively, while the lowest number of leaves was observed at the concentration of 400 mg/L ChNPs, with 1.95 leaves per shoot (Figure 3).

### 2.4. Effect of ChNPs on Chlorophyll and β-Carotene

**Figure 4:**
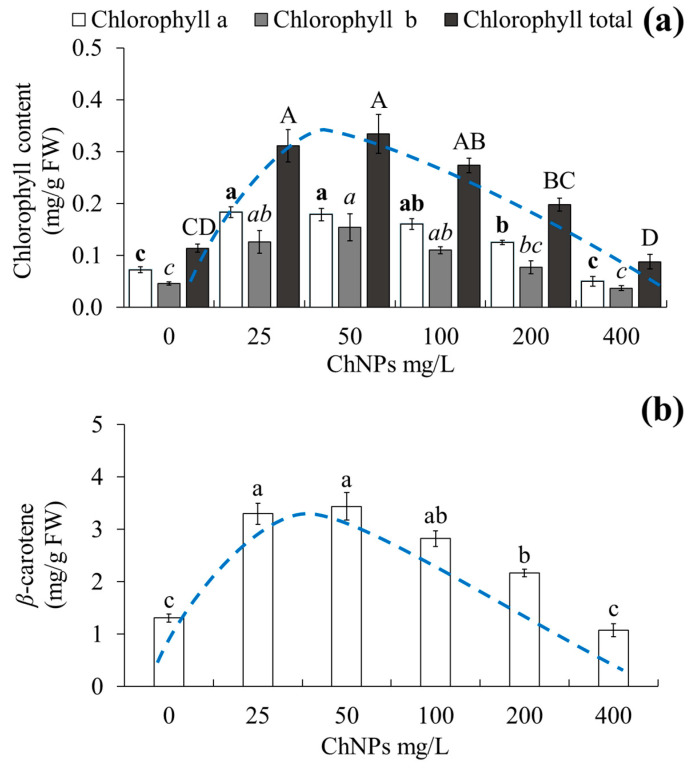
Photosynthetic pigment content after exposure of vanilla (*Vanilla planifolia* Jacks. ex Andrews) to different concentrations of ChNPs. (**a**) Chlorophyll content, (**b**) *β*-carotene after 60 days of culture. Data are expressed as mean values ± standard error (SE) to represent central tendency and variability. Means with a different letter indicate statistical significance (Tukey, *p* ≤ 0.05). Blue dashed line: hormetic dose–response curve depicting low-dose stimulatory (peak) and high-dose inhibitory responses (decline). ChNPs: chitosan nanoparticles, FW: fresh weight.

The administration of different ChNP concentrations had an effect on photosynthetic pigment contents (Figure 4). Chlorophyll concentrations showed significant differences among the treatments evaluated (Figure 4a). In relation to chlorophyll a, the greatest amount occurred with concentrations of 25 and 50 mg/L ChNPs, with 0.18 and 0.17 mg/g fresh weight, respectively, while the lowest amount was found at concentrations of control and 400 mg/L ChNPs, with 0.07 and 0.05 mg/g fresh weight, respectively. Regarding chlorophyll b, the greatest amount was found at concentrations of 25 mg/L ChNPs, with 0.15 mg/g fresh weight, while the lowest amount was obtained at concentrations of control and 400 mg/L ChNPs, with 0.04 and 0.03 mg/g fresh weight, respectively. The greatest total chlorophyll amount was found at concentrations of 25 and 50 mg/L ChNPs, with 0.31 and 0.33 mg/g fresh weight, respectively, while the lowest amount was obtained at concentrations of 400 mg/L ChNPs, with 0.08 mg/g fresh weight.

On the contrary, the greatest *β*-carotene amount was found at concentrations of 25 and 50 mg/L ChNPs, with 3.29 and 3.43 mg/g fresh weight, respectively, while the lowest amount was obtained at concentrations of control and 400 mg/L ChNPs, with 1.31 and 1.07 mg/g fresh weight, respectively (Figure 4b).

### 2.5. Effect of ChNPs on H_2_O_2_, MDA, Total Phenolic Content, and Total Antioxidant Capacity

**Figure 5:**
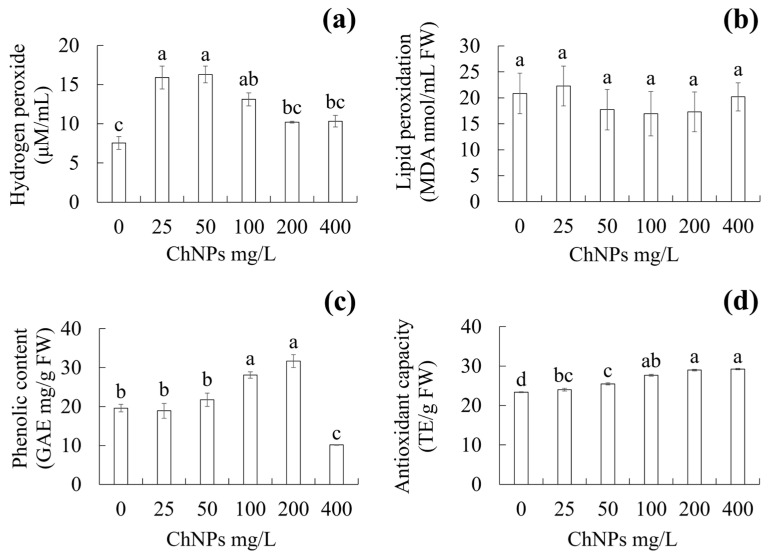
Effect of ChNPs on in vitro shoot proliferation of vanilla (*Vanilla planifolia* Jacks. ex Andrews). (**a**) Hydrogen peroxide, (**b**) lipid peroxidation expressed in malondialdehyde (MDA), (**c**) phenolic contents expressed in gallic acid equivalents (GAE), (**d**) antioxidant capacity expressed in trolox equivalents (TE), after 60 days of in vitro culture. Data are expressed as mean values ± standard error (SE) to represent central tendency and variability. Means with a different letter indicate statistical significance (Tukey, *p* ≤ 0.05). ChNPs: chitosan nanoparticles. FW: fresh weight.

The application of different chitosan nanoparticle concentrations had an effect on hydrogen peroxide, total phenolic content, and antioxidant capacity (Figure 5a,c,d). Regarding lipid peroxidation expressed in malondialdehyde (MDA), no significant differences were obtained among treatments (Figure 5b). For hydrogen peroxide content, the greatest level was obtained at concentrations of 25 and 50 mg/L ChNPs, with 15.90 and 16.29 µM/mL, respectively, while the lowest amount of hydrogen peroxide was observed in the concentration without ChNPs, at 7.54 µM/mL (Figure 5a). For total phenolic content, the greatest amount of GAE was obtained at concentrations of 100 and 200 mg/L ChNPs, with 28.1 and 31.69 GAE mg/g fresh weight, respectively, while the lowest amount of GAE was observed at a concentration of 400 mg/L ChNPs, with 10.15 GAE mg/g fresh weight (Figure 5c). As for total antioxidant capacity, the greatest trolox equivalent (TE) amount was found at concentrations of 200 and 400 mg/L ChNPs, with 25.00 and 24.46 TE/g fresh weight, respectively, while the lowest amount was found at the concentration without ChNPs, with 23.35 TE/g fresh weight (Figure 5d).

## 3. Discussion

### 3.1. Chitosan Nanoparticle Characterization

Physiochemical characterization of ChNPs was validated with SEM and FT–IR. These optical characteristics were found to be very similar to those found by Valentin et al. [21], Kou et al. [22], and Iswarya et al. [23]. Valentin et al. [21] reported that chitosan aerogel at 3435 cm^−1^ corresponds to the stretching vibration of the –NH_2_ and –OH functional groups. Asgari-Targhi et al. [24] reported that ChNPs at 3435 cm^−1^ and 3460 cm^−1^ correspond to the stretching vibration of the –NH_2_ and –OH functional groups.

### 3.2. Effect of ChNPs on Shoot Proliferation

This study demonstrated the effect of different ChNPs concentrations on the survival and development variables. The highest explant mortality was observed at concentrations of 100, 200, and 400 mg/L ChNPs. Toxicity of ChNPs at high concentrations has been described under in vitro conditions in species such as bell pepper (*Capsicum annuum*) [24], potato (*Solanum tuberosum* L.) [25], and milk vetch (*Astragalus* spp.) [26]. Asgari-Targhia et al. [24] found that 5, 10, and 20 mg/L ChNPs in *C. annum* inhibited growth during in vitro seed germination. Elsahhar et al. [25] reported that 300 mg/L ChNPs in *S. tuberosum* presented a low percentage response during shoot multiplication. Alhaithloul et al. [26] showed that 2 mg/L ChNPs in *Astragalus* spp. decreased the rooting percentage of shoots. The toxic effect of high ChNP concentrations on *V. planifolia* explants could be explained by a saturation of chitosan in the cells, which can lead to an imbalance in cellular homeostasis. In this regard, Alenazi et al. [27] point out that high concentrations of chitosan provoke high levels of reactive oxygen species (ROS) causing oxidation and death of the explants.

The number of shoots per explant, the shoot length, and the number of leaves increased at concentrations of 25 and 50 mg/L ChNPs, while inhibition was shown at concentrations of 200 and 400 mg/L ChNPs. This effect is known as hormesis. According to Bello-Bello et al. [12], the hormetic effect is characterized by stimulation at a low dose and inhibition at a high dose. The stimulation of the number of shoots per explant, shoot length, and number of leaves per shoot could be associated with the fact that ChNPs at low concentrations induce the synthesis of phytohormones such as auxins and cytokinins [28] and/or the stimulation of antioxidant enzymes through nitric oxide and hydrogen peroxide signaling pathways [29].

Biostimulation of growth at low ChNP concentrations has been described during in vitro conditions in species such as bell pepper (*Capsicum annuum*) [24] and milk vetch (*Astragalus* spp.) [26]. Asgari-Targhi et al. [24] found an increase in root length and total leaf area in *C. annuum* at 1 mg/L ChNPs. Alhaithloul et al. [26] reported an increase in rooting percentage, number of shoot roots, and root length in *Astragalus* spp. at 0.5 mg/L ChNPs.

### 3.3. Total Chlorophyll and Carotenoid Content

Photosynthetic pigments are required for photomixotrophic effects. Photomixotrophism is the ability of the explant to obtain metabolic energy from the culture medium and as a product of photosynthesis [30]. The different ChNP concentrations had different changes on photosynthetic pigment content. The increase in the levels of chlorophyll and *β*-carotene content at low concentrations of 25 and 50 mg/L ChNPs can be attributed to an improvement in the photosynthetic process because ChNPs at low concentrations contribute to the increase in the photosynthetic rate [31]. Under ex vitro conditions, an increase in chlorophyll amount with ChNPs has been reported in crops such as caspian sage (*Salvia abrotanoides*) [32], bean (*Phaseolus vulgaris*) [27], and garden thyme (*Thymus vulgaris*) [33]. Attaran Dowom et al. [32] found an increase in total chlorophyll in *S. abrotanoides* with 90 mg/L ChNPs. Alenazi et al. [27] reported an increase in chlorophyll a and b content in *P. vulgaris* with 5 mg/L ChNPs. Haghaninia et al. [33] reported an increase in total chlorophyll content in *T. vulgaris* with 10 mg/L ChNPs.

ChNPs do not participate directly in photomixotrophic processes but could could contribute indirectly through the synthesis of chlorophyll. The amino group of ChNPs can be an important source of nitrogen for metabolism. According to Attaran et al. [32], ChNPs stimulate the expression of genes involved in chlorophyll synthesis, enhancing the availability of free amino compounds released from chitosan, and may enhance photosynthetic efficiency by suppressing ethylene, which improves Rubisco activation.

Regarding carotenoid content, an increase in the amount of these pigments has been reported with ChNPs in crops such as caspian sage (*Salvia abrotanoides*) [32]), bean (*Phaseolus vulgaris*) [27], and broccoli (*Brassica oleracea* var. *italica*) [34]. Attaran Dowom et al. [32] found an increase in carotenoid content in *Salvia abrotanoides* with 90 mg/L ChNPs. Alenazi et al. [27] found an increase in carotenoid content in *Phaseolus vulgaris* with 5 mg/L ChNPs. Yousuf et al. [34] found an increase in carotenoid content in *B. oleracea* var. *italica* with 20 mg/L ChNPs.

Qu et al. [35] point out that the increase in chlorophyll and *β*-carotene may be due to chitosan-enhancing photosystem II activity and inhibition of the production of reactive oxygen species. These mechanisms suggest that chlorophyll and *β*-carotene content increases and/or is maintained through inhibition of its degradation. The decrease in photosynthetic pigment content at high ChNP concentrations could be due to a toxic effect.

### 3.4. Effect of ChNPs on Biochemical Parameters

Hydrogen peroxide (H_2_O_2_). H_2_O_2_ is a toxic ROS at high concentrations and could damage cells; however, at low levels, ROS can play important roles in some biological processes [36]. Hydrogen peroxide at low concentrations can act as a signaling molecule and secondary messenger, which activates physiological and biochemical processes and the antioxidant system [37,38]. The results suggest that the increase in H_2_O_2_ content at low ChNP concentrations prepares or warns against future lethal stress from high ChNP concentrations, while the decrease in hydrogen peroxide content with respect to increasing ChNP concentrations may be related to permanent cell damage, which could explain the low survival rate at high ChNP concentrations. Stancill and Corbett [39] state that there are enzymes such as superoxide dismutase (SOD), catalase (CAT), peroxidase (POD) and/or glutathione peroxidase (GPX) which help regulate hydrogen peroxide, generating an adequate redox balance for cell development. Taghizadeh et al. [40] observed a reduction in H_2_O_2_ content in zarroo (*Dracocephalum polychaetum* Boiss) during in vitro conditions with 150 mg/L ChNPs. ChNPs have been shown to help reduce oxidative stress by neutralizing hydrogen peroxide in plants under ex vitro conditions, such as in periwinkle (*Catharanthus roseus*) [41] and bean (*Phaseolus vulgaris*) [27]. Hassan et al. [41] obtained a reduction in H_2_O_2_ content in *C. roseus* during plantlet growth with 10 mg/L ChNPs. Alenazi et al. [27] found a reduction in H_2_O_2_ content in *P. vulgaris* during plantlet growth with 50 mg/L ChNPs. Hormesis induces genetic, physiological, and biochemical modifications. Whenever an explant is exposed to abiotic stress, it experiences oxidative stress that leads to the excessive generation of ROS, affecting cellular homeostasis. The disruption of homeostasis results in the expression of genes involved in detoxification and scavenging, chaperonin function, kinases, and protein phosphorylation, increasing the synthesis of plant hormones such as salicylic acid, jasmonic acid, and ethylene [12]. However, not only are ROS being generated but reactive nitrogen species could also be generated, which are signaling molecules occurring in the response to abiotic stress, such as nitric oxide (NO) and nitric dioxide (NH_2_), among others [42].

Lipid peroxidation. Lipid peroxidation is a damaging process in which free radicals attack lipids in cell membranes and other lipid-containing structures. Lipid peroxidation is an indicator of oxidative stress [43]. Malondialdehyde (MDA) is a three-carbon dialdehyde byproduct of lipid peroxidation, used as an indirect biochemical indicator of lipid peroxidation levels in plants [44]. In this work, no significant differences in MDA content were found between the ChNP treatments. This could be because the concentrations of ChNPs evaluated in this study do not affect the mechanism of lipid peroxidation. Hassan et al. [41] and Ali et al. [45] found that the MDA content in vinca (*C. roseus*) remained the same as the control treatment with 10 mg/L ChNPs. The insignificant difference in MDA probably occurred due to the culture time and the explant sampling site; future analyses could suggest longer culture times and test other sampling sites, such as stems and/or roots.

Phenolic content. Phenolic compounds are characterized as a group of small molecules with the ability to scavenge free radicals [46], improve development by promoting molecular signaling through compounds such as phenolic acid [47], and act on plant defense mechanisms by preventing oxidative damage [48]. The increase in TPC at 100 and 200 mg/L ChNPs could be attributed to mechanisms for protecting antioxidants. According to Kuljarusnont et al. [49], phenolic compounds such as the flavonoids myricetin, quercetin, and kaempferol have high antioxidant activity. Kumar et al. [50] state that during oxidative stress phenolic compounds can help mitigate oxidative damage in plants generated by ROS. On the contrary, the decrease in TPC at a concentration of 400 mg/L ChNPs could be attributed to a phytotoxic effect that resulted from the overabundance of ChNPs, causing tissue damage and death and thereby limiting the synthesis and/or degradation of phenolic compounds. This fact could explain the low survival rate of the explants at this concentration. The total phenolic content with ChNPs has been studied in ex vitro conditions in species such as wheat (*Triticum aestivum*) [51], bell pepper (*Capsicum annuum*) [52], and umbrella plant (*Schefflera arboriecola*) [53]. Hajihashemi and Kazemi [51] observed an increase in total phenolic content in *T. aestivum* with 50 mg/L ChNPs. Mawale and Giridhar [52] reported an increase in total phenolic content in *C. annuum* with 100 mg/L ChNPs.

Antioxidant capacity. DPPH antioxidant capacity refers to a substance’s ability to reduce or neutralize the DPPH free radical [54]. Estimating the amount of antioxidant capacity during in vitro culture is important to understand the role of antioxidant molecules in regulating free radicals. The trolox equivalent (TE) is used to determine antioxidant capacity [55]. The increase in TAC may be related to the production of enzymatic (SOD, CAT, POD, GPX, among others) and non-enzymatic (ascorbic acid, tocopherol, glutathione, among others) antioxidant compounds that help neutralize free radicals. In this study, no antioxidant enzymes were evaluated; however, according to Asomadu et al. [56], TAC estimates antioxidant capacity by the individual components of a biological system. Future studies suggest the evaluation of specific antioxidant enzymes such as SOD, CAT, POD, GPX, among others. Under in vitro conditions, Mancilla-Álvarez et al. [57] found an increase in antioxidant capacity in *Saccharum* spp. during their multiplication with 200 mg/L ChNPs. The antioxidant capacity with ChNPs has been studied in lettuce (*Lactuca sativa*) [58] and sea lily (*Pancratium maritimum*) [59]. Ramírez-Rodríguez et al. [58] obtained an increase in antioxidant capacity in *L. sativa* during germination with 0.1, 0.2, 0.4, and 0.8 mg/L ChNPs. According to Hassan et al. [41], ChNPs induce the antioxidant enzyme system by mitigating stress symptoms and scavenging H_2_O_2_. Rudenko et al. [60] state that enzymatic and non-enzymatic antioxidants help reduce ROS in different parts of the cell such as the chloroplasts, cell wall, cytosol, and vacuoles.

**Figure 6:**
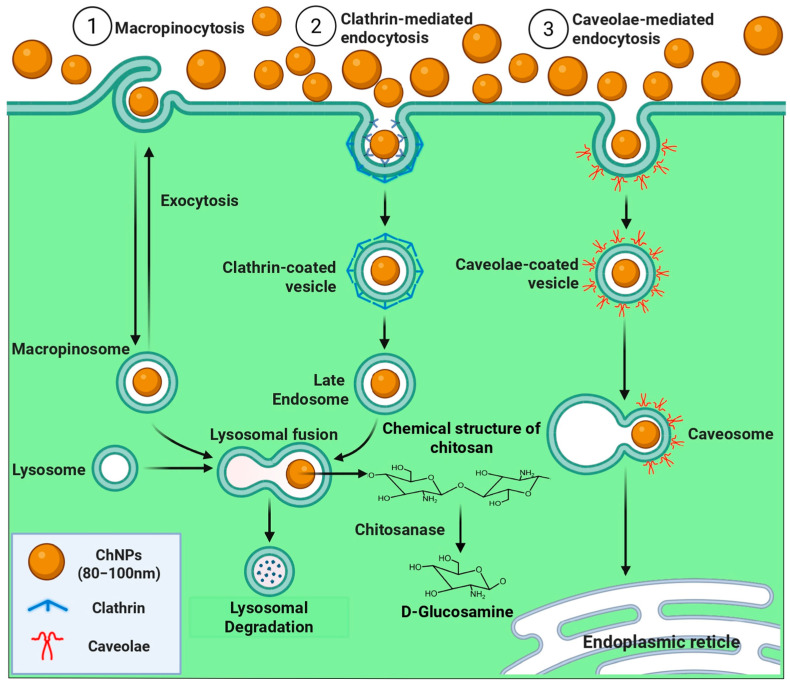
Internalization pathways of ChNPs through the plant cell membrane. ChNPs: chitosan nanoparticles.

According to Khan et al. [61], nanoparticles, in relation to their size, can be internalized into plant cells via aquaporins (up to 1.00 nm in size), cell wall pores (up to 10 nm in size), plasmodesmata (up to 40 nm in size), and endocytosis (up to 80 nm in size). In this study, according to the size of the ChNPs used, the internalization pathways suggest endocytosis through the plasma membrane. Endocytosis is the bioprocess by which cells internalize molecules, particles, materials, or membrane components through the formation of vesicles. There are different endocytosis pathways: macropinocytosis is a process in which the cell incorporates large volumes of extracellular fluid along with the molecules in suspension, forming large internal vesicles called macropinosomes; caveolae-regulated endocytosis, which is cholesterol-dependent and occurs at specific sites, and clathrin-mediated endocytosis, which controls receptor uptake in response to external signals (Figure 6). This study contributes to clarifying some uses of ChNPs in in vitro culture and recommends evaluating different NP sizes, enzymatic analyses, and reactive nitrogen species. Future studies on the mechanisms of uptake and intercellular movement of ChNPs in relation to their size are recommended. One possible mechanism for ChNP externalization or degradation is through exocytosis and/or hydrolysis to glucosamine monomers. Low glucosamine concentrations likely stimulate growth and activate defense mechanisms against a suspected fungal attack.

The different ChNP concentrations resulted in a hormetic effect on the development variables and chlorophyll and *β*˗carotene contents. The effects of ChNPs may be related to their physicochemical properties, such as their purity, aerodynamic particle size, molecular weight, form factor, solubility, and synthesis method. These characteristics can influence the effect that ChNPs have on plant tissues [62]. Hormesis is characterized by its biphasic response, which begins with a stimulus at a low concentration of a stressor and ends with an inhibition at a high concentration of the stressor agent. For ChNPs at high concentrations, studies suggest that toxicity depends on the nanoparticle size and deacetylation degree [28]. ROS play an important role during hormesis; at low concentrations, they act as signaling molecules that regulate growth, development, and tolerance to abiotic factors, while at high concentrations they can be toxic, causing cellular damage, impacting growth and differentiation, and resulting in cell death [63]. Increased H_2_O_2_ can inactivate the production of antioxidant enzymes and disrupt redox homeostasis [64]. Administration of ChNPs at appropriate concentrations could benefit growth and cell differentiation of different species during the multiplication stage. This study demonstrated that at low concentrations ChNPs can help mitigate oxidative stress by regulating H_2_O_2_ levels under in vitro conditions.

## 4. Materials and Methods

### 4.1. ChNPs Characterization

The physicochemical characteristics of ChNPs (NANOCHEMAZONE^®^, Leduc, AB, Canada) used in this study are shown in Table 1. The nanoparticles were characterized using a scanning electron microscope (SEM) (JEOL JSM 6360LA, Akishima City, TYO, Japan). The as-formed powder was suspended in high-purity water (Milli-Q) under sonification and was then a few drops of the mixture were dropped on the surface of the TEM carbon grid. The loaded grid was allowed to dry before being subjected to analysis. The photographs were taken using an electron beam with a power of 20 kV and a range of magnification of ×5000 to ×30,000.

### 4.2. Fourier Transform Infrared Spectroscopy (FT–IR)

The FT–IR spectroscopy method was determined according to Perea-Flores et al. [65]. An FT–IR spectrum was obtained using a Perkin Elmer^®^ FT–IR spectrophotometer (model Spectrum GX-100, Waltham, MA, USA) at room temperature with a universal attenuated total reflectance (ATR) in the spectral range of 4000 to 500 cm^−1^ and with a resolution of 4 cm^−1^ and 10 scans per sample.

### 4.3. Plant Material, In Vitro Culture and Incubation Conditions

The mother plant was collected at the Postgraduate College-Campus Cordoba, at the following geographical coordinates: 18°53′56.5″ N 96°56′58.9″ W. Cuttings were collected from 60 cm long vanilla containing five axillary buds each. Leafless stems were then washed for 10 min with running water and 2 drops of Tween 20^®^ (Merck KGaA^®^, Darmstadt, Germany). Nodal segments approximately 4–5 cm long were kept refrigerated at 5 ± 2 °C for 12 h. The nodal segments were then immersed in 0.25% (*v*/*v*) sodium hypochlorite solution for 5 min in a laminar airflow cabinet. Finally, the explants were immersed in a 5.5 µM mercuric chloride (HgCl_2_) solution for 15 min and rinsed four times with distilled water. The explants were reduced to 2 cm in length and cultured in test tubes (22 mm × 150 mm) containing 15 mL of MS medium [66] supplemented with 8.8 µM 6-benzylaminopurine (BAP, PhytoTech Labs, Lenexa, KS, USA), 4.9 µM indobutyric acid (IBA, Merck KGaA^®^), and 87.64 mM sucrose. The culture medium was adjusted to a pH of 5.7 ± 0.2 and 0.25% Gelrite^®^ (Merck KGaA^®^) was added. The medium was autoclaved at 1.03 bar at 120 °C for 20 min. The explants were incubated at 24 ± 1 °C under a 16:8 (light:dark) photoperiod with a photosynthetic photon flux density of 50 µmol m^−2^ s^−1^. After 21 days of in vitro culture, the explants were moved to the shoot proliferation stage on semisolid MS medium containing 2 mg/L BAP.

### 4.4. Effects of ChNPs on Shoot Proliferation

Bioreactors with a volume of 1 L were used; ten explants were added to each TIB. The TIBs consisted of twin flasks with a capacity of 500 mL, one with the explants and the other with the liquid culture medium. The flasks are connected with silicone hoses and use hydrophobic filters to prevent contamination as described by Escalona et al. [67]. The TIBs contained liquid MS medium supplemented with 87.64 mM sucrose and 8.8 µM BAP, and different concentrations of ChNPs were added: control, 25, 50, 100, 200, and 400 mg/L; acetic acid (JT Baker, Phillipsburg, NJ, USA) at 1% *v*/*v* was used to dissolve the ChNPs. The concentrations of the ChNPs were determined according to the following in vitro studies: Asgari-Targhi et al. [24] reported in bell pepper (*C. annuum*) concentrations of 5, 10, and 20 mg/L ChNPs. Elsahhar et al. [25] reported in potato (*S. tuberosum* L.) concentrations of 100, 200, 250, and 300 mg/L ChNPs. Alhaithloul et al. [26] reported in milk vetch (*Astragalus* spp.) concentrations of 0.2, 0.5, 1, 2, 3, and 4 mg/L ChNPs. The ChNPs were obtained by the Synthesis Electrostatic spray method (Electrospraying) as indicated in the technical data sheet. Four TIBs were used for each concentration and the whole experiment was performed in triplicate (four repetitions replicated three times = twelve repetitions in total). In the TIBs, the immersion frequency was 2 min every 4 h, as proposed by Ramos-Castellá et al. [68]. The compressor used in the TIBs maintains a constant air renew with atmospheric gases (O_2_, CO_2_, N_2_, among others). After 60 days of culture, the development variables, survival (%), and biochemical parameters were evaluated.

### 4.5. Quantification of Photosynthetic Pigments

Chlorophyll (Chl) estimation. Chlorophyll content was determined using the methodology described by Harborne [69]. To prepare the samples, 250 mg of fresh plant material was weighed and macerated by adding 2.5 mL of 70% acetone. Samples were stored at 5 ± 2 °C for 12 h. The samples were then filtered and adjusted to 6.25 mL with 70% acetone. Chlorophyll a and b content was determined in 3 mL aliquots by measuring absorbances of 663 and 645 nm, respectively, using a spectrophotometer (Lambda 35, Perkin-Elmer, Waltham, MA, USA) by adding a 70% acetone blank.

Total chlorophyll was calculated according to the following:

Chlorophyll a = Chl a (mg g^−1^) = 12.25* × λ663 nm − 2.79* × λ646 nm

Chlorophyll b = Chl b (mg g^−1^) = 21.50* × λ646 nm − 5.10* × λ663 nm

Chlorophyll total = Chl (a + b) (mg g^−1^) = 7.15* × λ663 nm + 18.71* × λ646 nm

λ: wavelength selected in a spectrophotometer

*: coefficient of equations

Carotenoid estimation. For carotenoid (*β*-carotene) content, the methodology described by Saini et al. [70] was followed and an absorbance of 450 nm was established for sample readings using a spectrophotometer (Lambda 35, Perkin-Elmer, Waltham, MA, USA). After 60 days of culture, photosynthetic pigment content was evaluated.

Carotenoids = (1000* × λ470 nm − 1.82* × Chl a− 85.02* × Chl b)/198

λ: wavelength selected in a spectrophotometer

*: coefficient of equations

### 4.6. Evaluation of Reactive Oxygen Species (ROS)

ROS measurement was performed using a colorimetric assay that measures hydrogen peroxide as a reactive oxygen metabolic byproduct. The Peroxide Assay kit MAK311-1KT (Merck KGaA^®^, Darmstadt, Germany) was used. First, the samples were deproteinized by taking 25 mg of leaf tissue ground with perchloric acid (PCA). The mixture was centrifuged at 3800× *g* at 8 °C for 15 min, then 500 µL of the supernatant was collected to add 1700 µL of reagent A, followed by 17 µL of reagent B for stirring. Subsequently, 20 µL of the supernatant was taken and 100 µL of reagents A and B were added and left to stand for 25 min. Finally, absorbances were read at 585 nm in a spectrophotometer (Lambda 35, Perkin-Elmer, Waltham, MA, USA).

### 4.7. Determination of Lipid Peroxidation

A colorimetric kit (Chemical^®^, Cambridge, UK) was used to determine lipid peroxidation expressed in malondialdehyde (MDA). First, 25 mg of leaf tissue was weighed and macerated, followed by the incorporation of both 375 µL of distilled water with 7.5 µL of butylated hydroxytoluene (C_15_H_24_O) for each sample and 1 vol of 2 N perchloric acid (HClO_4_). Then the extract was centrifuged at 3800× *g* at 8 °C for 20 min and the supernatant (150 µL) was collected and mixed with 2 mL of 20% trichloroacetic acid (C_2_HCl_3_O_2_) and 2 mL of 0.5% thiobarbituric acid (C_4_H_4_N_2_O_2_S). The mixture was heated at 90 °C for 35 min in a gas exhaust hood and later cooled on ice. The absorbance was read at 532 nm using a spectrophotometer (Lambda 35, Perkin-Elmer, Waltham, MA, USA).

### 4.8. Phenolic Content

Phenolic content was determined according to Payet et al. [71]. First, 250 mg of plant material was macerated with 5 mL of 70% methanol and then centrifuged at 3800× *g* at 8 °C for 20 min. The supernatant (0.150 mL) was then mixed with 0.75 mL of 10% Folin–Ciocalteu reagent (Merck KGaA^®^) and allowed to stand for 3 min, after which 0.6 mL of 20% sodium carbonate (Na_2_CO_3_) (Merck KGaA^®^) was added; the sample was then left to stand in the dark for 2 h. Phenolic content was determined by absorbance at 765 nm using a spectrophotometer (Lambda 35, Perkin-Elmer, Waltham, MA, USA).

### 4.9. Total Antioxidant Capacity (TAC)

TAC was determined according to Oxygen-Radical Absorbance Capacity (ORAC) essay. ORAC was formulated as DPPH (2,2-diphenyl-1-picrylhydrazyl) and was obtained using the methodology described by Burits and Bucar [72]. A 0.25 g sample was macerated with 2500 µL of 70% methanol. Then, the supernatant (~110 µL) was mixed with 2900 µL of DPPH and left to stand for 2 h. DPPH was expressed as trolox equivalent (TE) and a trolox standard solution was used for the calibration curve. Finally, it was determined at an absorbance of 515 nm using a spectrophotometer (Lambda 35, Perkin-Elmer, Waltham, MA, USA).

### 4.10. Data Analysis

Four replicates were used in a completely randomized experimental design. All statistical analyses were conducted using IBM SPSS Statistics, version 29. After 60 days of culture, the development variables and biochemical parameters were evaluated. The data were analyzed with analysis of variance (ANOVA), with Tukey’s honestly significant difference test (*p* ≤ 0.05).

## 5. Conclusions

In conclusion, the results demonstrated the hormetic effect of ChNPs during in vitro propagation of vanilla. It is suggested that application of ChNP concentrations in the range of 25 and 50 mg/L is optimal for stimulating growth and increasing photosynthetic pigment content, while concentrations above 100 mg/L have negative effects, leading to reduced survival rates and significant inhibition of morphological development. The application of ChNPs in micropropagation represents a novel approach to biostimulating development during the multiplication stage; however, it is recommended to apply these nanoparticles at different stages of micropropagation to evaluate their potential in other plant species.

## Figures and Tables

**Table 1:** Physicochemical characteristics of chitosan nanoparticles (ChNPs).

Product	ChNPs
Chemical Abstracts Service Registry Number	9012-76-4
Purity	>99.9%
Aerodynamic Particle Sizer	80–100 nm
Formula	C_6_H_11_NO_4_
MW	161 g/mol
Form Factor	Powder
Solubility	Soluble dilute aqueous acid
Synthesis method	Electrostatic spray

MW: molecular weight.

## Data Availability

Data will be made available on request.
